# Relevance of the two-component sensor protein CiaH to acid and oxidative stress responses in *Streptococcus pyogenes*

**DOI:** 10.1186/1756-0500-7-189

**Published:** 2014-03-28

**Authors:** Ichiro Tatsuno, Masanori Isaka, Ryo Okada, Yan Zhang, Tadao Hasegawa

**Affiliations:** 1 Department of Bacteriology, Nagoya City University Graduate School of Medical Sciences, 1 Kawasumi Mizuho-cho Mizuho-ku, Nagoya 467-8601, Japan

## Abstract

**Background:**

The production of virulence proteins depends on environmental factors, and two-component regulatory systems are involved in sensing these factors. We previously established knockout strains in all suspected two-component regulatory sensor proteins of the *emm1* clinical strain of *S. pyogenes* and examined their relevance to acid stimuli in a natural atmosphere. In the present study, their relevance to acid stimuli was re-examined in an atmosphere containing 5% CO_2_.

**Results:**

The *spy1236* (which is identical to *ciaH*_*py*_) sensor knockout strain showed significant growth reduction compared with the parental strain in broth at pH 6.0, suggesting that the Spy1236 (CiaH_py_) two-component sensor protein is involved in acid response of *S. pyogenes*. CiaH is also conserved in *Streptococcus pneumoniae*, and it has been reported that deletion of the gene for its cognate response regulator (*ciaR*_*pn*_) made the pneumococcal strains more sensitive to oxidative stress. In this report, we show that the *spy1236* knockout mutant of *S. pyogenes* is more sensitive to oxidative stress than the parental strain.

**Conclusions:**

These results suggest that the two-component sensor protein CiaH is involved in stress responses in *S. pyogenes*.

## Background

*Streptococcus pyogenes*, is a Gram-positive bacterium that infects the upper respiratory tract, including the tonsils and pharynx, and is responsible for post-infection diseases such as rheumatic fever and glomerulonephritis. *S. pyogenes* also causes severe invasive diseases including necrotizing fasciitis and streptococcal toxic shock syndrome (STSS) [1-5].

*S. pyogenes* is exclusively a human pathogen and it possesses many virulence factors that help it to resist host defense systems. The production of these factors is precisely regulated in response to host environmental conditions, such as different infection sites or host immune system induction levels [6-8]. In prokaryotes, the regulation of protein production in response to fluctuating environmental conditions depends primarily on two-component regulatory systems, which consist of a sensor histidine kinase and its cognate response regulator [9]. Thirteen two-component regulatory systems have been described in *S. pyogenes*, of which the CovRS system (also known as the CsrRS system) mediates the control of several virulence factors [10-15]. Analysis of the other two-component regulatory systems is still incomplete. In addition, most experiments have been performed from the viewpoint of the response regulators. Therefore, it is still unclear which signals the sensor proteins sense.

In a previous study, we focused on the sensor proteins of two-component regulatory systems, establishing 13 types of sensor knockout mutants, analyzing their involvement in the acid response in a “natural” atmosphere, and proposing that the Spy1622 two-component sensor protein is involved in sensing acid stimuli [16]. In contrast to the natural atmosphere used in our previous study, an atmosphere containing 5% CO_2_ is often used to culture *S. pyogenes*[10,17,18]. The CO_2_ concentration in deeper tissues is higher than its concentration at the epithelial surface of the host [19]. This can cause certain genes—for example, the gene encoding M protein—to be stimulated by carbon dioxide [20]. Therefore, it is possible that the genes involved in the acid response are also stimulated differently under natural atmospheric conditions than they are in an atmosphere containing 5% CO_2_. In the present study, we reanalyzed the involvement of 13 sensor proteins in the acid response in an atmosphere containing 5% CO_2_.

## Methods

### *S. pyogenes* strains

**Figure 1:**
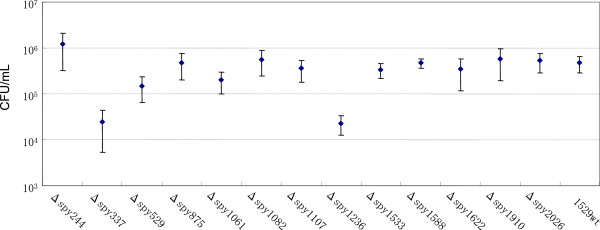
**Analysis of the growth of 13 sensor knockout strains cultured in acidic media (pH 6.0) in an atmosphere containing 5% CO**_**2**_**.** The CFU/ml after 23 h, broth culture of wild-type strain 1529 and its derived sensor knockout strains are shown. Viable counts were performed on BHI-Y and sheep blood agar plates. At least three independent experiments were performed. The error bars indicate the standard error of the mean (SEM).

Streptococcal strains 1529, MDYK, and MDN were isolated from Japanese patients with STSS [21,22]. *S. pyogenes* (GAS) strain SF370, which is currently the most prevalent database reference isolate (accession number NC_002737), was provided by J. J. Ferretti [23,24]. As shown in Figure 1, 13 sensor knockout mutants derived from the strain 1529 have previously been constructed [16]. These strains were cultured in either brain–heart infusion (E-MC62, EIKEN Chemical Co., Tokyo, Japan) supplemented with 0.3% yeast extract (BD, Sparks, MD, USA) broth (BHI-Y), or Todd Hewitt broth (BD) supplemented with 0.3% yeast extract broth (TH-YE), unless otherwise stated.

### Culture conditions for growth assay

Streptococcal strains were cultured using a previously described strategy [16], with certain modifications. In brief, an aliquot of frozen bacterial stock solution that had been stored at −80°C was inoculated into the TH-YE broth and cultured overnight (about for 18 h) at 37°C without agitation. A 70 μL sample of the overnight culture was added to fresh TH-YE broth (4 mL, pH 7.6 or 6.0), cultured in an atmosphere containing 5% CO_2_ for 23 h, and then the viable cells were counted by plating onto blood agar and BHI-Y agar plates. The experiments were repeated at least three times, independently.

### Production of *spy1236* knockout strains

We constructed an *S. pyogenes* strain 1529Δ*spy1236* as described previously [16]. Strains MDYKΔ*spy1236* and MDNΔ*spy1236* were constructed using the same strategy. To construct a plasmid for *spy1236* complementation (pLZ-spy1236), the DNA fragment was amplified using oligonucleotide primers 1236-n2 (5^′^-GTGGTTGACTTAGCTCGAAA-3^′^) and 1236-c2 (5^′^-AAAATTCATTGAACCTACAC-3^′^), strain 1529 genomic DNA as template, and PrimeSTAR HS DNA polymerase (Takara, Ohtsu, Japan). Digestion with *Pvu*II produced a fragment containing *spy1236*, which was treated with T4 polynucleotide kinase and ligated into the *Sma*I site of the plasmid pLZ12-Km2 [25].

### BLAST analysis

The Basic Local Alignment Search Tool (BLAST) was used for homology search (http://blast.ncbi.nlm.nih.gov/).

### The sensitivity of Δ*spy1236* mutants and derivative strains to H_2_O_2_

Assays were performed as described previously [26]. In brief, aliquots of bacterial cultures grown to an OD_660_ of ~0.3 were exposed to 61 mM H_2_O_2_ for 15 min at room temperature. Viable cells were counted by plating onto blood agar and BHI-Y agar plates before and after exposure to H_2_O_2_, and the result was expressed as percent survival.

### Plasmids having *htrA* gene

Plasmids pLZ-htrA_forward_ and pLZ-htrA_reverse_ were constructed as described in Additional file 1: Figure S1. In brief, a DNA fragment encoding the *htrA* gene was amplified using oligonucleotide primers htrA-F3 (5^′^-CATTACTTTTTACACAATTTATCCACAAGT-3^′^) and htrA-R1 (5^′^-GTAGGTCTATCAATAATTCTTTTGTCATAA-3^′^), strain1529 genomic DNA as template, and *TaKaRa Ex Taq* DNA polymerase (Takara). The PCR product was cloned into the pGEM®-T Easy vector (Promega, Madison, WI, USA). The resulting plasmid was digested with *Eco*RI and ligated into the *Eco*RI site of the plasmid pLZ12-Km2 [25] to yield pLZ-htrA_forward_ and pLZ-htrA_reverse_. In pLZ-htrA_forward_ and pLZ-htrA_reverse_, the *htrA* genes were cloned in opposite directions.

### Quantitative RT-PCR (qRT-PCR)

Total RNA was extracted from bacterial cells grown as described above for the H_2_O_2_ sensitivity assay. The purity and concentration of the RNA were determined by gel electrophoresis and spectrophotometry, respectively. Extracted total RNA was employed as the template for random-primed first-strand cDNA synthesis using a High Capacity cDNA Reverse Transcription Kit with RNAase Inhibitor (Applied Biosystems, Darmstadt, Germany) according to the manufacturer’s instructions. It was also used without reverse transcription, as a control to assess genomic DNA contamination. The cDNA and the control were then used as templates for quantitative RT-PCR (qRT-PCR) (real-time 7900HT PCR machine; Applied Biosystems) using the Sybr green detection system (Applied Biosystems). Primers for the genes of interest and the internal control gene *gyrA* are shown in Table 1. PCR conditions included incubation at 50°C for 2 min, followed by incubation at 95°C for 10 min, and finally 40-cycles of amplification (95°C for 15 s and 60°C for 1 min). The signal was standardized to that of the *gyrA* gene, where the cycle threshold (CT) was determined automatically using a real-time 7900HT PCR software (Applied Biosystems) after 40 cycles. Changes in the levels of gene expression were calculated using the ΔΔC_*T*_ method [27,28]. Each assay was repeated using at least three independent RNA samples. Product specificity was evaluated using both melting-curve analysis [29] and 2% agarose gels.

**Table 1:** Sequences of primers used in qRT-PCR

**Primer**	**Sequence (5**^**′**^**-3**^**′**^**)**
GyrA-1584 F	ACGTGGCGTCCAAGGAACT
GyrA-1709R	TGCTAAGCTTTCAACCGATAGACA
HtrA-F4	ATCGACGGAGCTAAACGAATTG
HtrA-R4	TCAGCTCCAACTAATTCACCAACA
Nrd-F	ACAGTAGACAAGCTGAAGACGGC
Nrd-R	AGCGAGTATGACACTGTTCACATTC
Emm1-31 F	TGCTACTCCAGCTGTTGCCATA
Emm1-98R	ACAGGTGAAACAGCTAACCCATTC
MGAS5005 polA-F	GCGGGCAAAACCACCTT
MGAS5005 polA-R	GCGCGACCCGCCTTATA

Primers were designed by using Primer Express® software version 3.0 and 2.0, and with technical support of life technologies Japan.

### Statistical analysis

Survival times were analyzed by using nonparametric Mann–Whitney U analysis and unpaired *t* test. *P* values of <0.05 for both analyses were considered statistically significant.

## Results and discussion

### Analysis of the effect of sensor proteins on the growth of bacteria cultured at pH 7.6 or 6.0

To test the effect of *S. pyogenes* sensor proteins on growth under acidic pH conditions, we first used the previously established knockout mutants lacking all 13 suspected sensor proteins [16]. We cultured the parental and derived knockout strains in a medium with the pH adjusted to 6.0, and in an atmosphere containing 5% CO_2_. As shown in Figure 1 and Table 2, the CFU (colony forming units)/ml for overnight cultures of strain 1529Δ*spy337* (*covS*) and 1529Δ*spy1236* were lower than that of the parental strain 1529. However, the CFU/ml for overnight cultures of strain 1529Δ*spy1622* was not lower than that of the parental strain 1529 under this experimental condition.

**Table 2:** **Growth of *spy1236 *** **knockout mutant in acidic (pH 6.0) media in an atmosphere containing 5% CO**_**2**_

**Strains**	**OD**_**660**_	**Av. CFU/mL**
**(pH7.6)**		
1529wt	0.906 ± 0.009	3.4 ± 0.7 × 10^8^
1529Δ*spy1236*	0.927 ± 0.025	2.0 ± 0.6 × 10^8^
**(pH6.0)**		
1529wt	0.662 ± 0.009	4.1 ± 1.6 × 10^5^
1529Δ*spy1236*	0.571 ± 0.039	2.3 ± 1.0 × 10^4^

The experiment was performed as described in Figure 1. At least three independent experiments were performed and they always yielded essentially the same results. Values expressed are the means (±SEM).

In this study, we focused on Spy1236 more than CovS, for the following three reasons. First, we have already analyzed 1529Δ*covS* and have showed that 1529Δ*covS* had lower growth ability than the parental wild type strain 1529 even at pH 7.6 [17]. Therefore, the lower growth ability of the *covS* mutated strain shown in Figure 1 may not have been caused by the fact that the medium was adjusted to pH 6.0. Second, CFU/ml (or OD_660_) for overnight cultures of strain 1529Δ*spy1236* was similar to that of the parental strain 1529, when cultured in a medium with pH adjusted to 7.6 and in an atmosphere containing 5% CO_2_ (Table 2). Finally, BLAST analysis showed that Spy1236 (436 amino acids) shares 58% identity with CiaH of *Streptococcus mutans* (referred to as CiaH_m_). It is known that the CiaH_m_ sensor kinase is involved in a response to acid stress in *S. mutans*[27,30,31].

**Figure 2:**
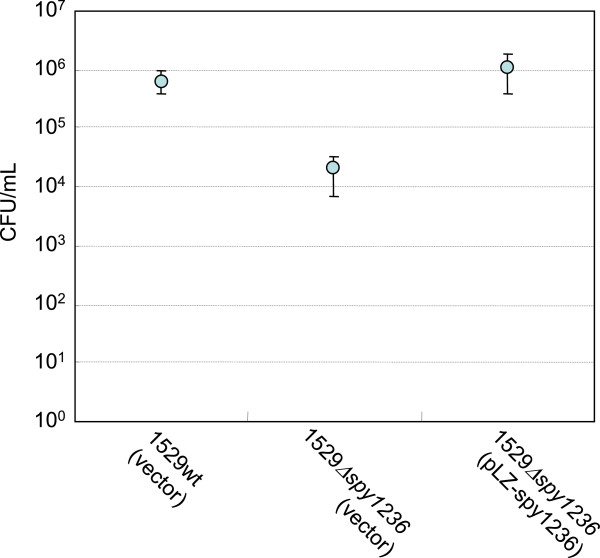
**Analysis of the growth of wild-type, *spy1236 *knockout, and complemented strains cultured in acidic (pH 6.0) media in an atmosphere containing 5% CO**_**2**_**.** The CFU/ml after 23 h, broth culture of wild-type strain 1529 and its derived strains are shown. Viable counts were performed on BHI-Y and sheep blood agar plates. At least three independent experiments were performed and they always yielded essentially the same results. The error bars indicate the standard error of the mean.

In order to further investigate the effect of Spy1236 sensor kinase on growth under acidic conditions, we next established a strain in which the s*py1236* knockout was complemented using an appropriate plasmid, and performed the same experiments at pH 6.0 and 5% CO_2_. As shown in Figure 2, *spy1236* cloned into a plasmid vector (pLZ-spy1236) complemented the lower growth ability of 1529Δ*spy1236*. These results suggested that the lower growth level of the *spy1236* mutant was Spy1236-dependent, at least in this strain.

**Figure 3:**
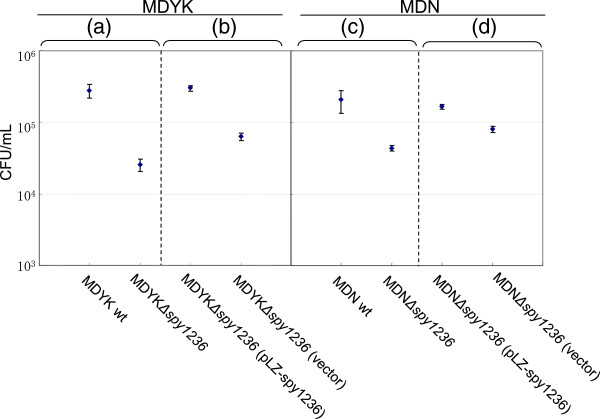
**Analysis of the growth of wild-type, *spy1236 *knockout and complemented strains cultured in acidic media (pH 6.0) in an atmosphere containing 5% CO**_**2**_**.** The experiment was performed as described in Figures 1 and 2. At least three independent experiments were performed and they always yielded essentially the same results. The error bars indicate the standard error of the mean. **(a)** The CFU/ml broth culture of wild-type strain MDYK and its derived strain MDYKΔ*spy1236* are shown. **(b)** The CFU/ml culture of MDYKΔ*spy1236* (pLZ-spy1236) and MDYKΔ*spy1236* (pLZ12-Km2) grown in a broth supplemented with 62.5 mg/mL kanamycin are shown. Viable counts were performed on BHI-Y agar plates supplemented with 125 mg/mL kanamycin. **(c)** The CFU/ml broth culture of wild-type strain MDN and its derived strain MDNΔ*spy1236* are shown. **(d)** The CFU/ml culture of MDNΔ*spy1236* (pLZ-spy1236) and MDNΔ*spy1236* (pLZ12-Km2) grown in a broth supplemented with 62.5 mg/mL kanamycin are shown. Viable counts were performed on BHI-Y agar plates supplemented with 125 mg/mL kanamycin.

To examine the effects related to strain specificity, we established additional *spy1236* knockout strains derived from strains MDYK and MDN (MDYKΔ*spy1236* and MDNΔ*spy1236*, respectively). When the same experiments were performed at pH 6.0 and 5% CO_2_, the CFU/ml for overnight cultures of MDYKΔ*spy1236* and MDNΔ*spy1236* were lower than those of the parental strains (Figures 3a and c). In addition, the CFU/ml for overnight cultures of MDYKΔ*spy1236* (pLZ-spy1236) and MDNΔ*spy1236* (pLZ-spy1236), in which the *spy1236* deletions were complemented with pLZ-spy1236, were higher than those for MDYKΔ*spy1236* (pLZ12-km2) and MDNΔ*spy1236* (pLZ12-km2), which harbor a control vector, respectively (Figure 3b and d). Thus, Spy1236 (also referred to as CiaH_py_) may be involved in the response to acid stress in some *S. pyogenes* strains, as it is in *S. mutans*, in an atmosphere containing 5% CO_2_.

Meanwhile, the empty-vector complementation resulted in the increased acid-resistance compared to the mutant strain (Figure 3). Kanamycin added to the complementation assay might induce some stress responses including the acid-resistance.

### Sensitivity of the *spy1236* knockout strains to oxidative stress

**Figure 4:**
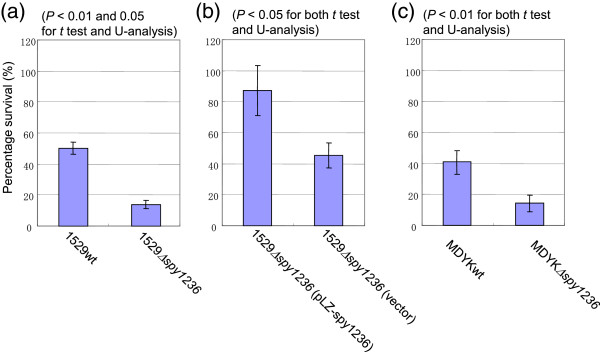
**H**_**2**_**O**_**2 **_**sensitivity assays for 1529**Δ***spy1236 *and MDYK**Δ***spy1236 *strains.** H_2_O_2_ sensitivity assays were performed for wild-type 1529 and 1529**Δ***spy1236* strains **(a)**, 1529**Δ***spy1236* (pLZ-*spy1236*) and 1529**Δ***spy1236* (pLZ-12-Km2) strains **(b)**, or wild-type MDYK and MDYK**Δ***spy1236* strains **(c)**. 1529Δ*spy1236* (pLZ-*spy1236*) and 1529Δ*spy1236* (pLZ-12-Km2) strains were grown in broth supplemented with 62.5 mg/mL kanamycin. H_2_O_2_ (61 mM) was added to 1-mL aliquots of culture grown to an OD_660_ of ~0.3. After 15 min at room temperature (~22°C), viable counts were performed on BHI-Y and sheep blood agar plates before and after the addition of peroxide, and the percentages of survival were calculated. The BHI-Y agar plates were supplemented with 125 mg/mL kanamycin for the 1529Δ*spy1236* (pLZ-*spy1236*) and 1529Δ*spy1236* (pLZ-12-Km2) strains. Values expressed are the means (± SEM) of three independent experiments.

The CiaH sensor kinase is also conserved in *Streptococcus pneumoniae* (51% identical with Spy1236 by BLAST analysis). Ibrahim *et al*. [26] showed that deletion of the gene encoding the cognate response regulator CiaR_pn_ made a pneumococcal strain more sensitive to oxidative stress. Therefore, we were interested to learn whether the Spy1236 sensor kinase is involved in the response to oxidative stress, and performed essentially the same experiments using *S. pyogenes* Δ*spy1236* mutants as were previously done using *S. pneumoniae*[26]*.* As shown in Figure 4a, 1529Δ*spy1236* was significantly more sensitive to hydrogen peroxide than the parental strain 1529, and the complemented strain 1529Δ*spy1236* (pLZ-spy1236) was more resistant to hydrogen peroxide than 1529Δ*spy1236* (pLZ12-Km2, the control vector) (Figure 4b). To examine the effects related to strain specificity, we also performed this experiment in strain MDYK. The knockout strain MDYKΔ*spy1236* was more sensitive to hydrogen peroxide than its parental strain, MDYK (Figure 4c). Thus, the CiaH_py_ sensor kinase of *S. pyogenes* may contribute to oxidative stress tolerance.

The percent survival of 1529Δ*spy1236* (pLZ12-Km2) (Figure 4b) seems to be similar to that of wild-type strain 1529 (Figure 4a). One of the differences in their experimental settings is that 1529Δ*spy1236* (pLZ12-Km2) was grown in broth supplemented with kanamycin. This might induce some stress responses to increase the survival rate of the 1529Δ*spy1236* (pLZ12-Km2). At least, we did not find a potential region to confer the ability, when the pLZ12-Km2 sequence was analyzed using BLAST.

### Contribution of the CiaH sensor kinase to oxidative stress tolerance may not be mediated via HtrA

**Figure 5:**
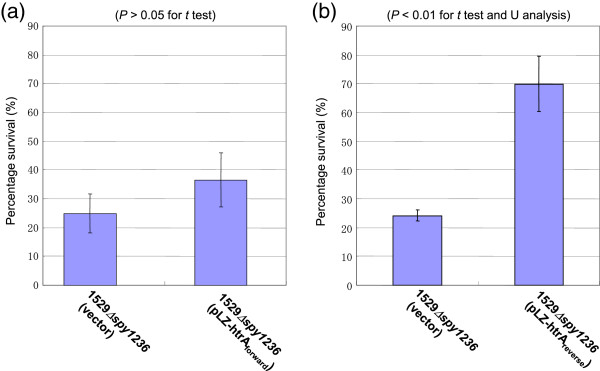
**H**_**2**_**O**_**2 **_**sensitivity assay for 1529**Δ***spy1236 *with the overexpression of *htrA*.** H_2_O_2_ sensitivity assays were performed for 1529**Δ***spy1236* (pLZ-*htrA*_forward_) **(a)** or 1529**Δ***spy1236* (pLZ-*htrA*_reverse_) strains **(b)**. Bacteria were grown in broth supplemented with 62.5 mg/mL kanamycin. H_2_O_2_ (61 mM) was added to 1-mL aliquots of culture grown to an OD_660_ of ~0.3. After 15 min at room temperature (~25°C), viable counts were performed as described in Figure 4. Values expressed are the means (± SEM) of three independent experiments.

Ibrahim *et al*. [26] also demonstrated that the contribution of the CiaH_pn_ sensor kinase to oxidative stress tolerance was mediated by the HtrA protein in *S. pneumoniae*, based on the following evidence: (i) the sensitivity of the *S. pneumoniae* strain D39Δ*ciaR* to oxidative stress can be restored by complementation with HtrA, and (ii) the expression of *htrA* in the CiaR-null mutant was down-regulated. HtrA, also known as DegP or DO protease [32], is a stress-induced serine protease that manifests both general molecular chaperone and proteolytic activities, and switches from chaperone to protease in a temperature-dependent manner [33]. HtrA is also conserved in *S. pyogenes* and is known to be essential for oxidative tolerance in *S. pyogenes*[34]. Therefore, we were interested to learn whether the contribution of the Spy1236 (CiaH_py_) sensor kinase to oxidative stress tolerance was also mediated by the HtrA protein in *S. pyogenes* (HtrA_py_). We first attempted to determine whether the sensitivity of the 1529Δ*spy1236* strain to oxidative stress could be restored by complementation with HtrA_py_; i.e., to investigate whether the first evidence shown in *S. pneumoniae* is also true in *S. pyogenes*. For this purpose, *htrA*_*py*_ of *S. pyogenes* was cloned into pLZ12-Km2 to yield pLZ-htrA_forward_ and pLZ-htrA_reverse_ (Additional file 1: Figure S1). The *htrA*_*py*_ gene is cloned into pLZ-htrA_reverse_ in the direction opposite to that in pLZ-htrA_forward_. When pLZ-htrA_forward_ was introduced into 1529Δ*spy1236,* the sensitivity of the resultant strain 1529Δ*spy1236* (pLZ-htrA_forward_) to hydrogen peroxide was not significantly different than that of 1529Δ*spy1236* (control vector) (Figure 5a). This may have resulted from the insufficient expression of HtrA_py_ by the pLZ-htrA_forward_ contained within 1529Δ*spy1236*. In addition, we hypothesized that level of HtrA expression from pLZ-htrA_reverse_ was greater than that from pLZ-htrA_forward_ (See Additional file 1: Figure S1 for detailed explanation). As shown in Figure 5 (b), 1529Δ*spy1236* having pLZ-htrA_reverse_ was more resistant to hydrogen peroxide than 1529Δ*spy1236* having pLZ12-Km2 (control vector). This result suggests that the overexpression of *htrA* may contribute the oxidative tolerance in that *spy1236* mutant.

**Figure 6:**
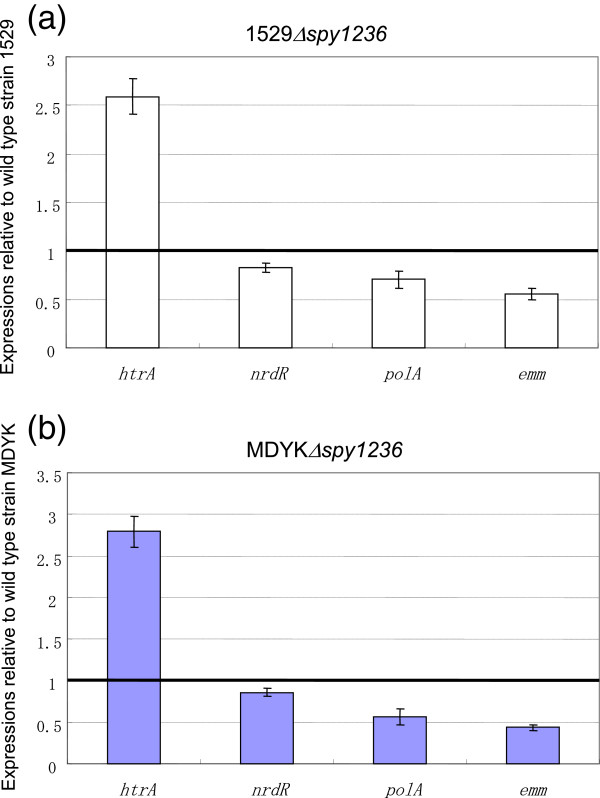
**Expression levels of Spy1236-regulated genes evaluated using qRT-PCR. (a)** Expression in 1529Δ*spy1236* mutant relative to that in the wild-type strain (1529). **(b)** Expression in MDYKΔ*spy1236* mutant relative to that in the wild-type strain (MDYK). Error bars represent the SEM of at least three biological repeats.

Next, we attempted to investigate the down-regulation of HtrA in the CiaH-null mutant; i.e., to determine whether the second evidence shown in *S. pneumoniae* is also true in *S. pyogenes*. For this purpose, expression of *htrA*_*py*_ was measured using qRT-PCR. Surprisingly, expression of *htrA*_*py*_ was not decreased in strains 1529Δ*spy1236* and MDYKΔ*spy1236,* compared with the parental strains 1529 and MDYK (Figure 6a and b). Therefore, we could not conclude that the contribution of the Spy1236 sensor kinase to oxidative stress tolerance was mediated by the HtrA protein in *S. pyogenes.* This result evoked further questions about what mediates control of oxidative stress tolerance by Spy1236 in *S. pyogenes.* In addition to HtrA, probably, there are at least two systems (NrdR- and PolA1-dependent, respectively) for oxidative stress tolerance in *S. pyogenes.* NrdR is a transcription factor first described in *Streptomyces coelicolor*[35] that regulates the expression of ribonucleotide reductase genes [36]. The ribonucleotide reductase genes are involved in the proliferation of *Salmonella* Typhymurium inside macrophages [37]. PolA1, a putative DNA polymerase I, has been reported to contribute to peroxide stress defenses in *S. pyogenes*[38]. Therefore, we measured the expression of *nrdR* and *polA1* (Figure 6a and b), and observed that their expression levels were slightly decreased in strains 1529Δ*spy1236* and MDYKΔ*spy1236,* compared with the parental strains (Figure 6a and b).

Thus, the contribution of Spy1236 (CiaH_py_) sensor kinase to oxidative stress tolerance may not be mediated via HtrA in *S. pyogenes* (Additional file 2: Figure S2). If this hypothesis is true, what factor mediates the contribution of Spy1236 to the tolerance? The slightly decreased expression of *polA1* (and/or *nrdR*) may be insufficient to explain why *spy1236* mutant that the expression of *htrA* is increased is lower than the parental wild type in the oxidative tolerance ability. Our next experiments will attempt to identify a Spy1236 regulon.

## Conclusions

In this study, we have demonstrated that the CiaH_py_ sensor kinase of *S. pyogenes* is involved in the response to acid and/or oxidative stresses, as are the related sensor kinases in *S. mutans* and/or *S. pneumoniae*. However, an important subject remains to be solved; i.e., it is still unclear how the CiaR/H_py_ two-component regulatory system is involved in the virulence of *S. pyogenes*, whereas the CiaR/H two-component regulatory systems in *S. mutans* and *S. pneumoniae* are already known to be involved in regulating virulence. At least using a mouse infection model, the virulence of Δ*spy1236* mutants is not significantly different from that of the parental *S. pyogenes* strains (Tatsuno *et al*., unpublished results). However the infection model seems to investigate the middle to late, but not the early stages of infection, because more than 10^7^ CFU of bacteria are inoculated into each mouse (10–12 g) [17], and this number is equivalent to >10^10^ CFU in a human. Therefore, the CiaH_py_ sensor kinase of *S. pyogenes* may not be important for virulence in the late stage of the infection, whereas it is still possible that CiaH_py_ confers some benefits to *S. pyogenes* in earlier infection stages, as proposed for the CovS sensor kinase in a previous study [17].

The present and previous studies suggested that CO_2_ condition is important for the triggering the function of Spy1236, but not of Spy1622 [16]. There are some helpful reports to discuss the potential mechanism about why CO_2_ is required for triggering Spy1236 regulatory function [39,40]. M1 and PrtF1/SfbI are both fibronectin binding proteins, which are required for *S. pyogenes* invasion of mammalian cells. PrtF1/SfbI expression is enhanced in an O_2_-rich environment, while M1 expression is greater at higher CO_2_ partial pressure [20,41]. It has been explained that the differential regulation of these two Fn-binding proteins in high O_2_ or high CO_2_ may allow *S. pyogenes* to adapt to several different *in vivo* environments, such as those on the skin, on mucosal surfaces, and within the tonsils. When *S. pyogenes* encounters acid stress at the epithelial surface of the host, the bacterium might need the expression of different genes, compared with the genes required when exposed to acid and/or oxidative stresses in deeper tissues. If this hypothesis is true, the Spy1236 regulon should be different from a Spy1622 regulon. Although CovR was already found to influence transcription of 15% of all chromosomal genes using DNA microarrays [42], such analysis has not been adopted for the other two-component systems in *S. pyogenes*. Our next experiments will attempt to identify the Spy1236 and the Spy1622 regulons.

Not only the present study but also previous studies have not ever determine whether CiaH directly senses acidic signal [26,27,30,31]. Although the established method to address this question does not exist as far as we know, if the phosphorylation status or regulatory activity of cognate response regulator, or the expression of Spy1236-regulated genes is demonstrated, they may provide some answers to the question.

### Availability of supporting data

There are three supporting data of Additional file 1: Figure S1, Additional file 2: Figure S2, and Additional file 3: Figure S3.

## Abbreviations

BHI-Y: Brain-heart infusion yeast; TH-YE: Todd Hewitt yeast.

## Competing interests

The authors declare that they have no competing interests.

## Authors’ contributions

IT conceived the study. IT, RO, and TH designed and performed the experimental work with help by YZ and MI. All authors contributed to the data analysis. IT wrote the original manuscript. TH helped to produce the final manuscript. All authors read and approved the final manuscript.

## Supplementary Material

Additional file 1: Figure S1 Schematic representations of the pLZ-htrA_forward_ and pLZ-htrA_reverse_ plasmids used for overexpression of the *htrA* gene in *S. pyogenes*. Click here for file

Additional file 2: Figure S2 Hypothetical working model for the response to oxidative stress mediated by the two-component system Spy1236-1237. HtrA is regulated by systems other than Spy1236 in *S. pyogenes*. Dotted arrows indicate hypothetical pathways. Click here for file

Additional file 3: Figure S3 Expression levels of *htrA* and *polA1* in 1529Δ*spy1236* (pLZ-htrA_reverse_) relative to those in 1529Δ*spy1236* (pLZ-htrA_forward_) evaluated using qRT-PCR. The expression of *htrA* in 1529Δ*spy1236* (pLZ-htrA_reverse_) was 1.5 (± 0.1) times that in 1529Δ*spy1236* (pLZ- htrA_forward_), while the expression of *polA1* in 1529Δ*spy1236* (pLZ-htrA_reverse_) was 0.94 (± 0.08) times that in 1529Δ*spy1236* (pLZ- htrA_forward_). Error bars represent the SEM of four experiments. Click here for file
