# Supplementary material for: Relevance of the two-component sensor protein CiaH to acid and oxidative stress responses in Streptococcus pyogenes
Source: BMC Res Notes. 2014 Mar 28;7:189. doi: 10.1186/1756-0500-7-189 (PMC3986815; doi:10.1186/1756-0500-7-189)
Supplement: Additional file 1: Figure S1 — Schematic representations of the pLZ-htrAforward and pLZ-htrAreverse plasmids used for overexpression of the htrA gene in S. pyogenes. [file 1756-0500-7-189-S1.ppt]

## Slide 1
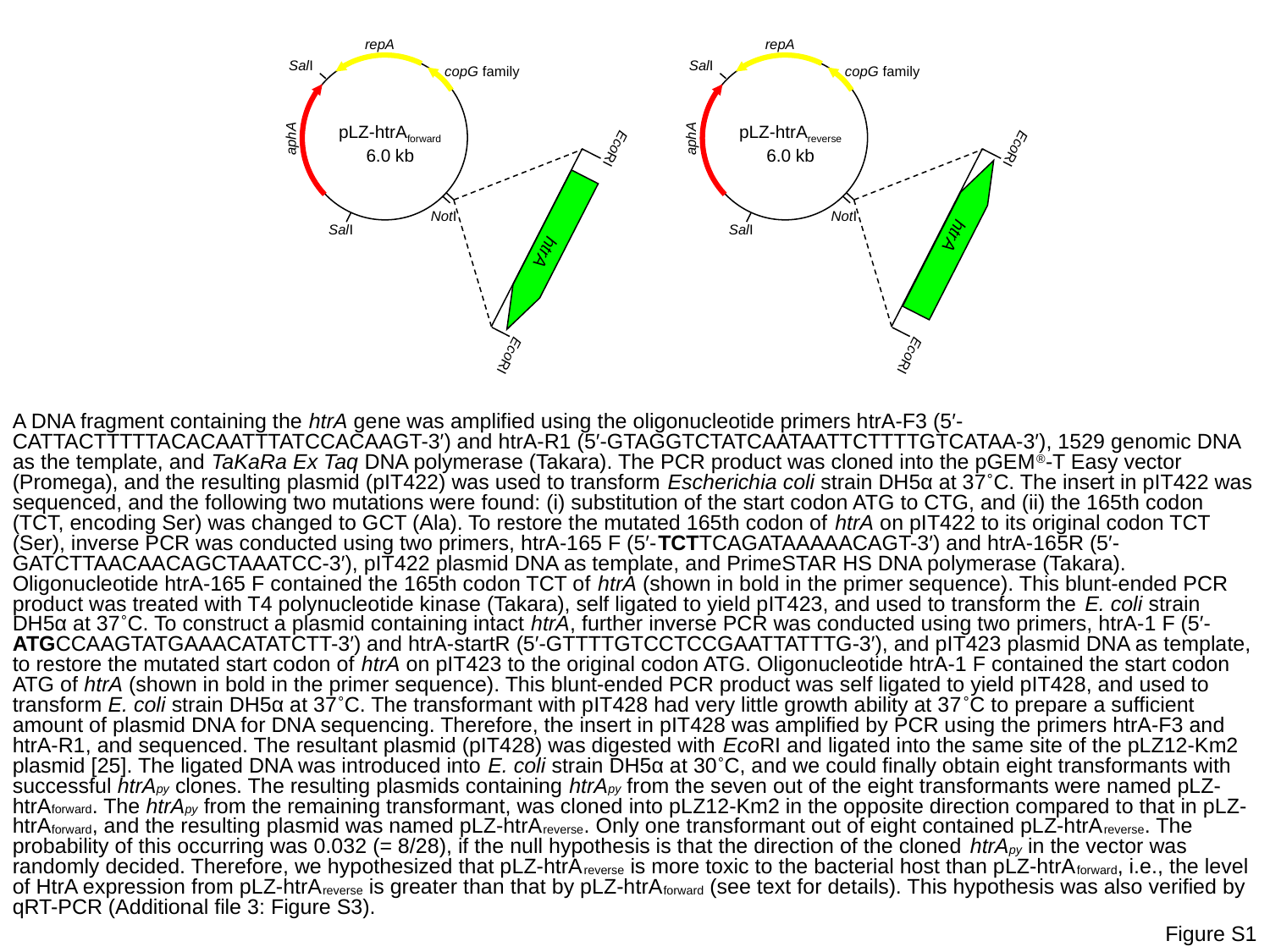

repA
repA
SalI
SalI
copG family
copG family
pLZ-htrAforward
6.0 kb
pLZ-htrAreverse
6.0 kb
aphA
aphA
EcoRI
EcoRI
NotI
NotI
SalI
SalI
htrA
htrA
EcoRI
EcoRI
# A DNA fragment containing the htrA gene was amplified using the oligonucleotide primers htrA-F3 (5′-CATTACTTTTTACACAATTTATCCACAAGT-3′) and htrA-R1 (5′-GTAGGTCTATCAATAATTCTTTTGTCATAA-3′), 1529 genomic DNA as the template, and TaKaRa Ex Taq DNA polymerase (Takara). The PCR product was cloned into the pGEM®-T Easy vector (Promega), and the resulting plasmid (pIT422) was used to transform Escherichia coli strain DH5α at 37˚C. The insert in pIT422 was sequenced, and the following two mutations were found: (i) substitution of the start codon ATG to CTG, and (ii) the 165th codon (TCT, encoding Ser) was changed to GCT (Ala). To restore the mutated 165th codon of htrA on pIT422 to its original codon TCT (Ser), inverse PCR was conducted using two primers, htrA-165 F (5′-TCTTCAGATAAAAACAGT-3′) and htrA-165R (5′-GATCTTAACAACAGCTAAATCC-3′), pIT422 plasmid DNA as template, and PrimeSTAR HS DNA polymerase (Takara). Oligonucleotide htrA-165 F contained the 165th codon TCT of htrA (shown in bold in the primer sequence). This blunt-ended PCR product was treated with T4 polynucleotide kinase (Takara), self ligated to yield pIT423, and used to transform the E. coli strain DH5α at 37˚C. To construct a plasmid containing intact htrA, further inverse PCR was conducted using two primers, htrA-1 F (5′-ATGCCAAGTATGAAACATATCTT-3′) and htrA-startR (5′-GTTTTGTCCTCCGAATTATTTG-3′), and pIT423 plasmid DNA as template, to restore the mutated start codon of htrA on pIT423 to the original codon ATG. Oligonucleotide htrA-1 F contained the start codon ATG of htrA (shown in bold in the primer sequence). This blunt-ended PCR product was self ligated to yield pIT428, and used to transform E. coli strain DH5α at 37˚C. The transformant with pIT428 had very little growth ability at 37˚C to prepare a sufficient amount of plasmid DNA for DNA sequencing. Therefore, the insert in pIT428 was amplified by PCR using the primers htrA-F3 and htrA-R1, and sequenced. The resultant plasmid (pIT428) was digested with EcoRI and ligated into the same site of the pLZ12-Km2 plasmid [25]. The ligated DNA was introduced into E. coli strain DH5α at 30˚C, and we could finally obtain eight transformants with successful htrApy clones. The resulting plasmids containing htrApy from the seven out of the eight transformants were named pLZ-htrAforward. The htrApy from the remaining transformant, was cloned into pLZ12-Km2 in the opposite direction compared to that in pLZ-htrAforward, and the resulting plasmid was named pLZ-htrAreverse. Only one transformant out of eight contained pLZ-htrAreverse. The probability of this occurring was 0.032 (= 8/28), if the null hypothesis is that the direction of the cloned htrApy in the vector was randomly decided. Therefore, we hypothesized that pLZ-htrAreverse is more toxic to the bacterial host than pLZ-htrAforward, i.e., the level of HtrA expression from pLZ-htrAreverse is greater than that by pLZ-htrAforward (see text for details). This hypothesis was also verified by qRT-PCR (Additional file 3: Figure S3).
Figure S1
