# Supplementary material for: Relevance of the two-component sensor protein CiaH to acid and oxidative stress responses in Streptococcus pyogenes
Source: BMC Res Notes. 2014 Mar 28;7:189. doi: 10.1186/1756-0500-7-189 (PMC3986815; doi:10.1186/1756-0500-7-189)
Supplement: Additional file 2: Figure S2 — Hypothetical working model for the response to oxidative stress mediated by the two-component system Spy1236-1237. HtrA is regulated by systems other than Spy1236 in S. pyogenes. Dotted arrows indicate hypothetical pathways. [file 1756-0500-7-189-S2.ppt]

## Slide 1
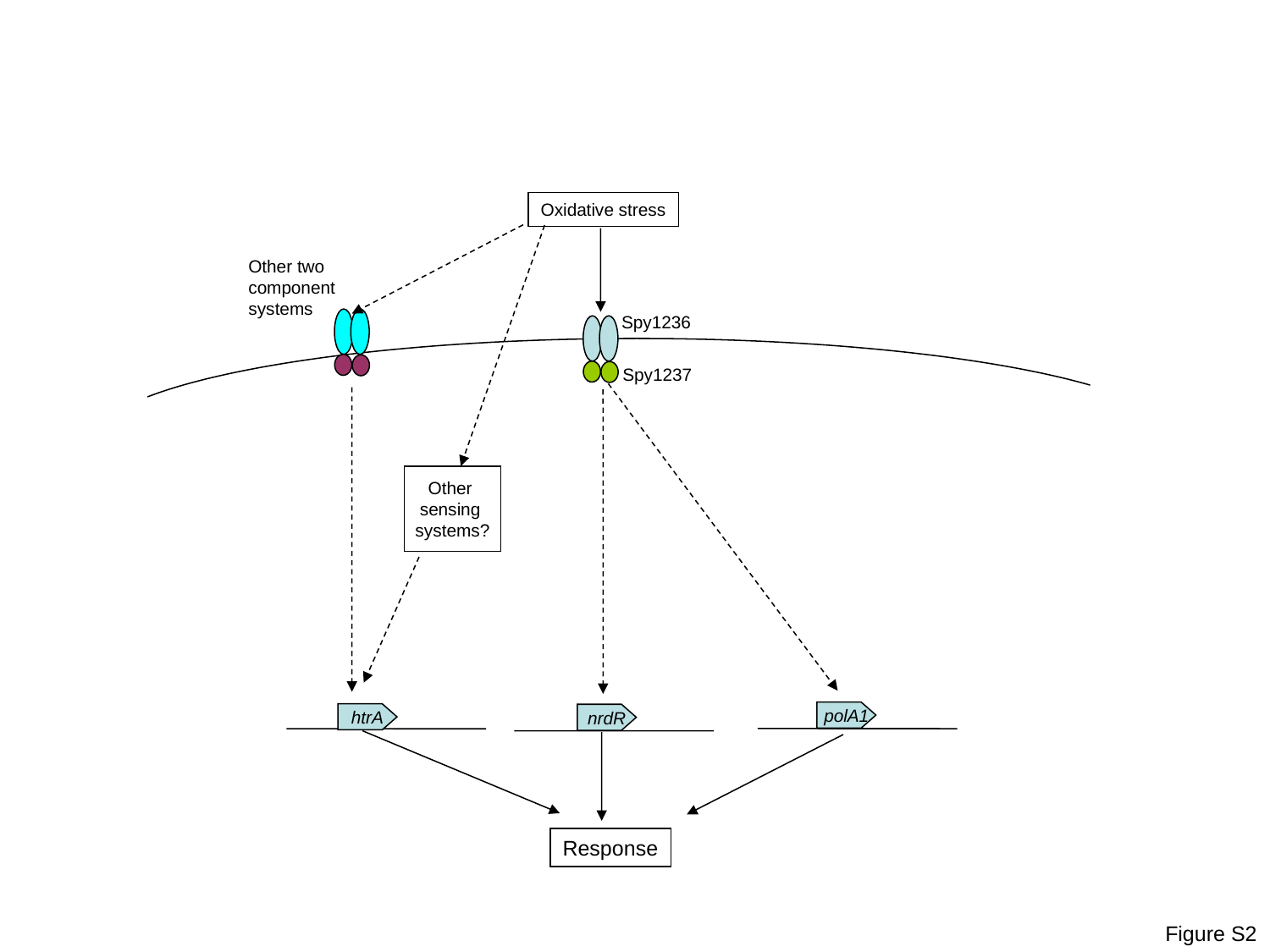

Oxidative stress
Other two component systems
Spy1236
Spy1237
Other
sensing
systems?
polA1
htrA
nrdR
Response
Figure S2
