# Supplementary figures and images for: Relevance of the two-component sensor protein CiaH to acid and oxidative stress responses in Streptococcus pyogenes
Source: BMC Res Notes. 2014 Mar 28;7:189. doi: 10.1186/1756-0500-7-189 (PMC3986815; doi:10.1186/1756-0500-7-189)

## Slide 1
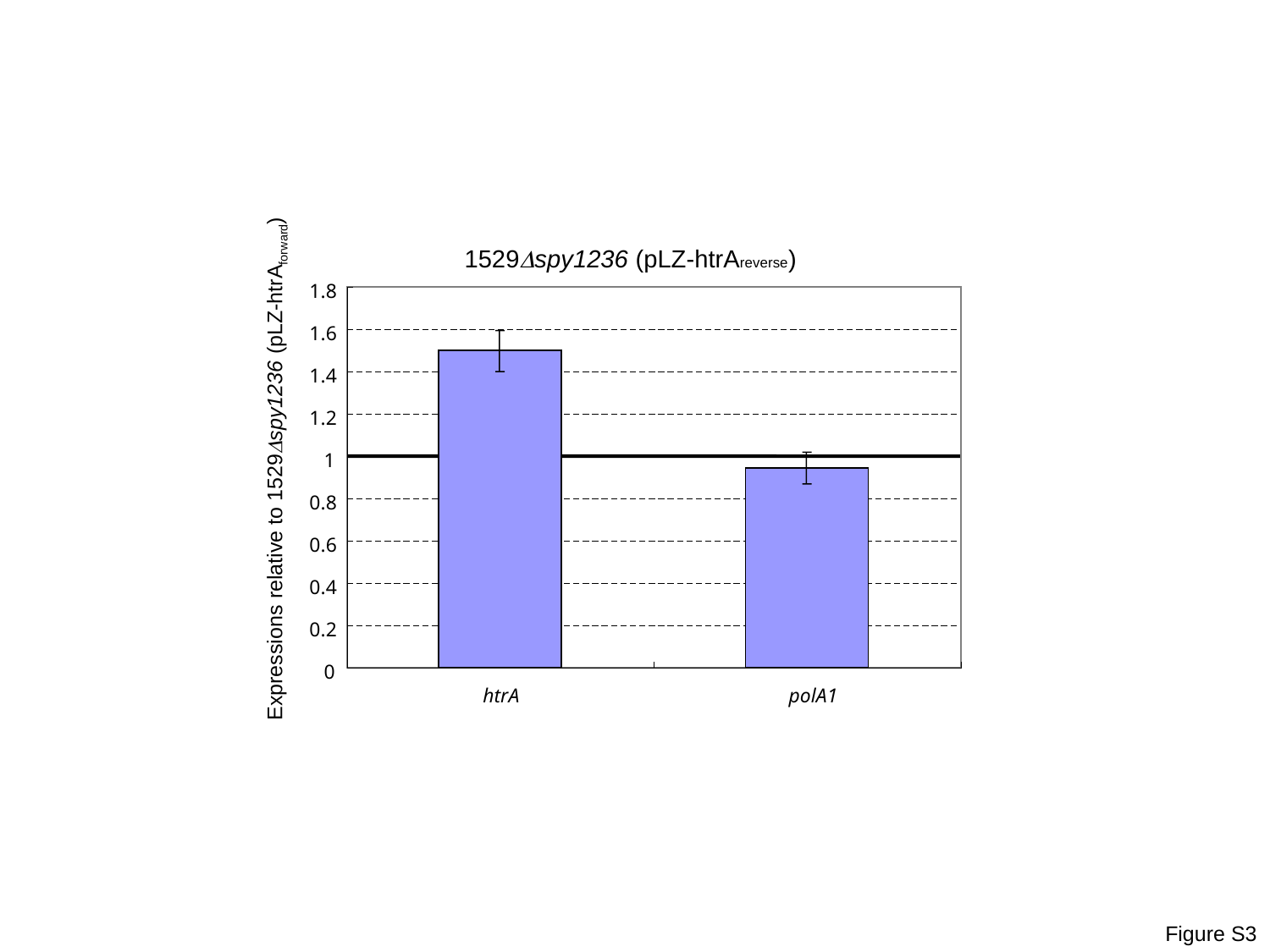

1529Δspy1236 (pLZ-htrAreverse)
1.8
1.6
1.4
1.2
Expressions relative to 1529Δspy1236 (pLZ-htrAforward)
1
0.8
0.6
0.4
0.2
0
htrA
polA1
Figure S3

Supplement: Additional file 3: Figure S3 — Expression levels of htrA and polA1 in 1529Δspy1236 (pLZ-htrAreverse) relative to those in 1529Δspy1236 (pLZ-htrAforward) evaluated using qRT-PCR. The expression of htrA in 1529Δspy1236 (pLZ-htrAreverse) was 1.5 (± 0.1) times that in 1529Δspy1236 (pLZ- htrAforward), while the expression of polA1 in 1529Δspy1236 (pLZ-htrAreverse) was 0.94 (± 0.08) times that in 1529Δspy1236 (pLZ- htrAforward). Error bars represent the SEM of four experiments. [file 1756-0500-7-189-S3.ppt]
